# VIPPrint: Validating Synthetic Image Detection and Source Linking Methods on a Large Scale Dataset of Printed Documents

**DOI:** 10.3390/jimaging7030050

**Published:** 2021-03-08

**Authors:** Anselmo Ferreira, Ehsan Nowroozi, Mauro Barni

**Affiliations:** Department of Information Engineering and Mathematics, University of Siena, 53100 Siena, SI, Italy; ehsan.nowroozi65@gmail.com (E.N.); barni@dii.unisi.it (M.B.)

**Keywords:** digital image forensics, source identification, GAN-generated image detection

## Abstract

The possibility of carrying out a meaningful forensic analysis on printed and scanned images plays a major role in many applications. First of all, printed documents are often associated with criminal activities, such as terrorist plans, child pornography, and even fake packages. Additionally, printing and scanning can be used to hide the traces of image manipulation or the synthetic nature of images, since the artifacts commonly found in manipulated and synthetic images are gone after the images are printed and scanned. A problem hindering research in this area is the lack of large scale reference datasets to be used for algorithm development and benchmarking. Motivated by this issue, we present a new dataset composed of a large number of synthetic and natural printed face images. To highlight the difficulties associated with the analysis of the images of the dataset, we carried out an extensive set of experiments comparing several printer attribution methods. We also verified that state-of-the-art methods to distinguish natural and synthetic face images fail when applied to print and scanned images. We envision that the availability of the new dataset and the preliminary experiments we carried out will motivate and facilitate further research in this area.

## 1. Introduction

The abundant availability of new technologies for generating physical documents such as printers and scanners has raised many concerns about their misuse, examples of which include generating illegal documents, misguiding investigations through the generation of fake evidence, or even hiding relevant evidence in criminal investigations. For instance, child pornography can be printed and distributed between pedophiles in order to avoid virtual monitoring from the police, and illegal amendments can be incorporated in printed contracts without previous notice. Furthermore, professional printers can be used to print fake currency and packages of fake products, causing several negative effects on the economy. Finally, printing and scanning can be used to hide the traces of image manipulation or the synthetic nature of the images, since the artifacts commonly found in manipulated, and synthetic images are not present or detectable after the images have been printed and scanned.

As a countermeasure to the diffusion of counterfeited printed documents, most of the major manufacturers of color laser printers have signed a secret agreement with governments to let the printers include secret (invisible) yellow dots onto printed documents [1]. Such dots, also called machine identification codes (MIC) or simply printer steganography, are used to identify the source of printed documents, as unique yellow-dots patterns are used to identify different printers. However, such a feature is not enabled in all laser printers, and as shown in [2], the yellow-dot patterns can be easily anonymized, leaving the authentication of printed documents problem unsolved.

The challenges posed by printed document forensics have pushed the multimedia forensics research community to look for viable solutions based on the analysis of the artifacts left by the printers on the printed documents. In general, printed document forensics can be split into three main research areas: (i) source linking (also known as printer attribution); (ii) detection of printed manipulated images; and (iii) detection of printed and scanned synthetic images. Solutions for printer attribution are mostly based on the analysis of the extrinsic artifacts contained in printed documents, with the most popular ones for laser printers being banding, jitter, and skewed jitters. The presence of these artifacts has been exploited by several works to identify the sources of printed texts [3,4,5,6,7,8,9,10,11,12,13,14,15,16,17,18,19,20,21,22], color images [23,24,25,26,27,28,29,30,31,32,33], or both [34,35,36]. Manipulation detection in printed documents has received some attention only recently [37] and mainly refers to unveiling post-processing operations that could alter the semantic meanings of the images. It usually exploits texture descriptors and deep neural networks to identify the visual artifacts introduced by such manipulations. Finally, as far as we know, despite the intense research devoted to the detection of images generated by generative adversarial networks (GANs) [38,39,40], scarce attention has been paid to the detection of such images in printed documents.

The research carried out so far notwithstanding, progresses in this area is hindered by the lack of large reference datasets. The few existing datasets, in fact, present at least one of the following issues: (i) they contain ad hoc data prepared for specific research only; (ii) the printed patterns are often simple ones, such as icons, text, and halftone patterns; (iii) most of them consider old and non-professional printers; (iv) they do not consider copies of the same printer brand and model; and (v) to the best of our knowledge, no dataset with complex fake printed images exists. This last issue is particularly important and challenging, as most of the artifacts used to detect image manipulations, such as the correlation between RGB channels, discrete cosine transform irregularities, and even illumination inconsistencies, are gone after the images are printed and scanned. This problem is worsened by the observation that printing and scanning back a manipulated image is one of the most powerful and simplest attacks an adversary can conceive of to fool manipulation detectors. The availability of a large reference dataset overcoming the above problems may be of great help to foster new advances in printed document investigations, concerning both the detection of manipulated and synthetic documents and the attribution of printed documents to the device that generated them.

In this paper, we aim at filling said gaps by presenting a large scale dataset that can be used for both applications: source attribution and synthetic image detection. Due to their relevance in image forensics applications, the dataset focuses on face images. In particular, the initial version of the dataset (we are planning to update it continuously in the next years) is composed of images printed by several printers and scanned back with a high-quality scanner. The images in the dataset are divided into (i) pristine images, to be used for the source attribution problem; and (ii) synthetic face images generated by three different generative adversarial networks (GANs). The dataset is further split into several subsets, containing regions of interest with different sizes for the investigation of localized artifacts. To evaluate the difficulties associated with the forensic analysis of the images contained in the dataset, we carried out an extensive comparative study including several source attribution and synthetic image detection baseline methods.

In summary, the contributions of this paper are:

1. We present a large scale dataset of color-printed face images for digital image forensics purposes, such as source attribution and synthetic images detection (deep fake images).
2. We increased the diversity of the images in our dataset to make it suitable for approaches working on images of different sizes. Full scanned images and regions of interest with different sizes are available.
3. To the best of our knowledge, our dataset is the first large scale dataset with printed and scanned artificial images created with GANs such as StyleGAN2 [41], ProgressiveGAN [42], and StarGAN [43].
4. We present the results of an in-depth comparative study conducted on the new dataset regarding several baseline approaches, including both data-driven methods and methods based on handcrafted features. The comparison regards both source attribution and synthetic image detection.

The rest of this paper is organized as follows: in Section 2 we report some related work and discuss the limitations of datasets used in the literature. In Section 3, we present our dataset and several configurations considered to generate data. In Section 4, we discuss the experimental setup considered to assess the difficulty of such a dataset. Finally, Section 5 reports the achieved results and, in Section 6, we conclude this paper and discuss the future work that we are aiming to do in such a dataset.

## 2. Related Work

Several works have investigated the exploitation of the artifacts left by the printers into the printed documents to identify their source. Here we focus our works aiming at source linking after document scanning, as they are usually cheaper, non-destructive, and fast.

Common surveys in the literature [44,45,46,47] divide source linking methods according to the kind of documents they focus on, namely: printed text documents, printed color image documents, or both. Moreover, we can distinguish between methods aimed at identifying the technology used to print the documents—inkjet, laser, etc.—and those trying to link the printed document to the single device that was used to print it [48,49,50,51,52]. In this section, we briefly review the second class of methods, since research in that area is more advanced.

Generally speaking, there are two kinds of clues in printed documents that could guide a forensic investigation aimed at identifying the specific source of the document: intrinsic and extrinsic signatures. Intrinsic signatures are introduced by the printing process itself, whereas extrinsic signatures are intentionally inserted into the printed material. Three of the most investigated intrinsic signatures in laser printers are banding, jitter, and skewed jitters. Eid et al. [24] characterized banding as a textural pattern composed of horizontal, low frequency, and periodic artifacts caused by the laser printer components variation, vibration, and speed regulation that can uniquely identify different printers. Similarly, the jitter consists of horizontal artifacts, but with a different frequency range and duration, and is caused by oscillatory disturbances of the printer’s drum and the developer roller. Finally, skewed jitter is also a periodic artifact like the others, but it differs from the previous ones as it is formed by vertical lines. With regard to extrinsic signatures, some relevant works include embedding code sequences in electrophotographic halftone images [53] and also machine identification codes [54]. Approaches based on extrinsic signatures require expensive modifications in the printing device, and also some of these extrinsic signatures can even be erased from the printed material [2].

With regard to the attribution of printed texts (i.e., black and white dots only), most of the techniques based on intrinsic signatures treat such a problem as a texture identification problem, as artifacts such as banding are not easy to be obtained from text [26]. For this set of techniques, the same patterns are extracted from the documents and subtle differences among them can be discriminated when printed by different printers [34]. One of the pioneers works in this regard comes from Ali et al. in 2004 [3]. The authors consider the pixel values of letters “I” as features in a multi-class classification problem. After the letters are classified, the source of a document can be found by verifying the most voted class among all the individual letters “I” classification. Several other techniques used a similar pipeline with few modifications, such as considering the statistics of gray-level co-occurrence matrices [4,5,6,11], Distance Transform [8], Discrete Cosine Transform [10], statistics of gray-level co-occurrence matrices together with residual noise and sub-bands of wavelet transform [12,13,14,17], deep neural networks [18,20], ad hoc texture descriptors [19,22] among others [7,9,15,16,21].

A second set of techniques to identify the source of any printed document focuses on the intrinsic signatures of documents containing colored pictures. In this case, banding artifacts are more evident as more patterns are printed (including the background). In this regard, one of the pioneer works is the one from Ali et al. [23], where the authors proposed to capture banding artifacts by applying the Fourier transform in image patches to get different banding frequencies. Eid et al. [24] applied a similar strategy to jitter artifacts by using Gabor filtering and discrete Fourier transform.

Another set of research on colored documents source linking treated intrinsic artifacts as noise. Choi et al. [26] discriminated printers by calculating 39 noise features from the diagonal (HH) sub-band of the discrete wavelet transform in pairwise and individual RGB and CMYK channels. In a subsequent work by the same authors [27], noise was estimated after Wiener filtering and gray level co-occurrence matrix statistics. Tsai et al. [29] calculated 45 statistics in HH, LH and HL sub-bands of a discrete wavelet transform with further feature selection. Choi et al. [30] extended their previous work in [26] by estimating noise with Wiener filtering and a 2D discrete wavelet transform, and characterizing it with 384 statistical filters on gray level co-occurrence matrices that described single channels of residual images and pair-wise channels. Other important techniques for color documents source attribution involve describing geometric distortions [25,32] and halftone texture descriptors [28,31,33].

Finally, a number of techniques aim at identifying the sources of printed documents regardless of their content. Ferreira et al. [34] proposed an extension of the gray level co-occurrence matrix descriptor considering more directions and scale and also a new descriptor, called convolutional texture gradient filter, that builds histograms of filtered textures with specific gradients intervals. The authors validated these approaches not only on the letter "E" of printed text, but also on regions of interest called frames, which are rectangular areas with sufficient printed material—images, text, or both. Bibi et al. [36] used a similar strategy using chunks of printed materials, but their solution involves convolutional neural networks. Finally, Tsai et al. [35] apply nine different filters, fusing several previous strategies such as extracting features from gray-level co-occurrence matrices, discrete wavelet transform, spatial filters, Gabor filter, Wiener filter, gray level co-occurrence matrices features, and fractal features.

The abundant development of source linking approaches for printed documents notwithstanding, the identification of image forgeries and synthetic images from a printed and scanned version of a digital image has received considerably less attention. One of the few works in this area has been published in [37], where simple print and scan attacks of manipulated printed documents with recompression, filtering, noise addition, and other simple image operations are detected by a specialized CNN architecture.

Therefore, although printed document forensics (especially printer source attribution) has received much attention in the last few years, there are still several issues to be tackled before solutions applicable to real-world scenarios are developed. Among them, the following two issues are relevant for the present paper:

1. There is a need for a publicly available dataset that grows through time to include modern printers with different technologies and manufacturing procedures. We expect that different printers manufactured at different times generate different artifacts in printed documents that cannot be detected by previous works.
2. There is a need for multimedia forensic techniques able to detect deepfake printed images. This is a very challenging problem, since several artifacts, such as the correlation between RGB channels, discrete cosine transform irregularities, and even illumination inconsistencies in the digital image versions are usually removed by the print and scan process. In this way, although several adversarial attacks have been discussed in the literature [55], the print and scan procedure is the easiest yet most powerful attack an adversary could perform against deepfake digital image detectors.

Therefore, the present work aims at moving a first step towards the solution of the above problems. This is done by presenting a long term dataset addressing both tasks: real-world source attribution with modern printers, and deepfake detection in printed and scanned documents. The details of the dataset we have constructed are described in the following sections.

## 3. The VIPPrint Dataset

In this work, we present a new dataset trying to minimize some of the issues of existing datasets. The new dataset, which we call VIPPrint (the dataset is named after the VIPP group), consists of two sections. The first one focuses on printer source attribution and solves some common limitations in previous works, such as (i) lack of diversity and (ii) lack of redundancy. Concerning the lack of diversity, the dataset contains printers of different models and printing resolutions. This is an important issue when considering source attribution in real-world applications such as anti-counterfeiting detection, where the printing resolution used for printing a counterfeited document or package is unknown. The inclusion in the dataset of diverse printers marks a significant difference concerning existing datasets, which usually look at artifacts associated with specific printing technologies at fixed resolutions. As to lack of redundancy, very few works have analyzed the effect of the presence of two or more printers of the same model and brand in the dataset, thus neglecting the overlapping effect associated with the presence of two identical printers. The second section of the dataset considers an important, yet understudied, problem in digital image forensics: the detection of synthetic fake images such as those created by Generative Adversarial Networks after print and scan.

The importance of the new dataset for digital image forensics is twofold: (i) it can foster the development of novel solutions for digital image forensics capable of withstanding a print and scan procedure, and (ii) it can inspire new techniques for source attribution of fake colored documents printed by modern printers, thereby linking the fake content to the owner, or user, of the printer.

Concerning the content of the images composing the dataset, we decided to consider face images. The first reason for such a choice is that face images are particularly relevant in many applications related to biometric recognition, criminal investigations, and misinformation. A second reason is the availability of large scale datasets of face images that can be used as a starting point for the construction of the printed and scanned dataset. Giving researchers the possibility to work both with the digital images and their printed and scanned versions can represent an added value in many applications. Finally, AI-based techniques to generate synthetic images are particularly advanced in the case of face images, whose quality has reached unprecedented levels with no or very few semantic artifacts the forensic analysis can rely on [41].

The details of the two sections the VIPPrint dataset consists of are discussed in the following.

### 3.1. VIPPrint Dataset for Source Attribution

**Figure 1:**
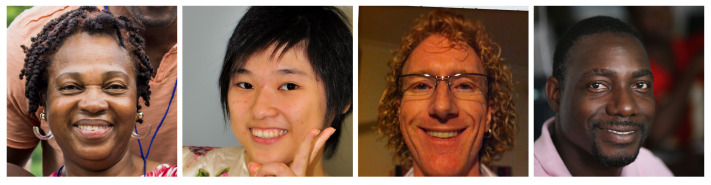
Some digital images considered from the work of Karras et al. [41] to build our dataset of printed images.

To select the images to print in the first dataset, we choose images from a dataset that has particular importance in the digital image forensics literature. These images come from the original subset of human faces from the Flickr-Faces-HQ (FFHQ) dataset [41]. We use these images for two reasons: (i) they have enough samples to generate a large dataset of printed images, which can be used by data-hungry techniques such as those based on deep learning; and (ii) they can be used to develop methods focusing on applications (e.g., child pornography) for which printing patterns usually found in other datasets (e.g., barcodes and text) are not useful. Some examples of the images included in the first section of the dataset are shown in Figure 1.

We choose to print the images in the dataset with printers that are diverse enough to make the source attribution problem challenging enough for state-of-the-art techniques. The initial version (As we said, we are planning to continuously update the dataset with new images, printed with other printers.) of such sub-dataset for source attribution contains 1600 printings from the printers listed in Table 1. We would like to highlight the difficulties associated with such a dataset as it contains modern printers, with some of them being professional laser printers that were commercialized in the last five years. The dataset also contains printers with different printing resolutions: for example, printers #1 and #8 have native resolutions different from the others (600 × 600 dpi).

The printers used were available in normal conditions (that means they were not exclusively used to print our dataset). Most of the printers were realtively new (they were from weeks to years old), and some of them needed toner replacements while printing. As for the scanner, we used the scanner from the Kyocera TaskAlfa3551ci multifunctional printer (printer #3 in Table 1), with 600 × 600 dpi scanning resolution. Moreover, we used the default sharpness for scanning and images are saved in a lossless compression configuration. As shown in Table 1, we printed 200 images per printer. For that, we used 50 A4 sheets of paper, printing four images per sheet using the landscape orientation, and then extracting individual patches.

**Figure 2:**
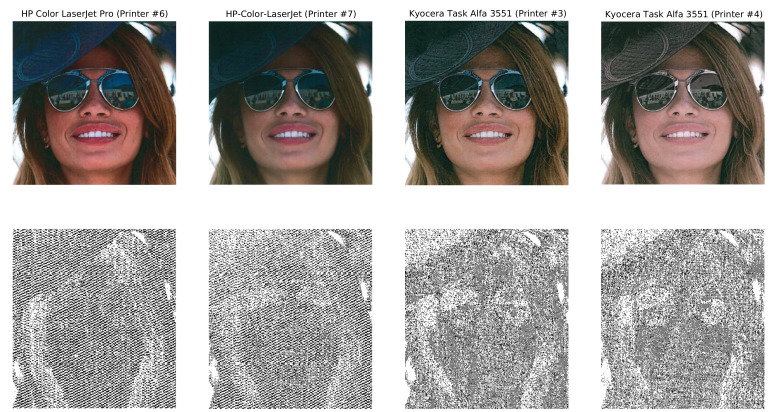
The same image (193.jpg) printed by four different printers and their corresponding HH discrete wavelet transform subbands (luminance component).

To illustrate the difficulties associated with source-linking of the images in the dataset, in Figure 2 we show the same image printed by different printers and its HH DWT subbands, which were used by Choi et al. [26] to perform source attribution of colored documents. Very subtle differences can be seen in HH subbands of different printers from the same brand but different models (Printers #6 and #7 in Figure 2), but no clear differences in the HH subband when using the same brand and model (Printers #3 and #4 in Figure 2).

As we were aware that 200 images per printer may not be enough for data-hungry techniques such as those based on deep learning, we produced a second set of images containing Regions of Interest (ROI) extracted from the full images set. The importance of the ROI sub-dataset for classification algorithms is three-fold: (i) it may filter only areas that are useful for recognition (e.g, areas containing edges); (ii) such areas can be input to techniques that require lots of data such as data-driven approaches; and (iii) they allow the classification of documents through the fusion of their ROIs classification, providing the most accurate results. Such a strategy was validated several times before in the digital forensics domain, such as in works for camera source attribution [56,57], anti-spoofing solutions [58] and other works in laser printer source attribution [34,36].

**Figure 3:**
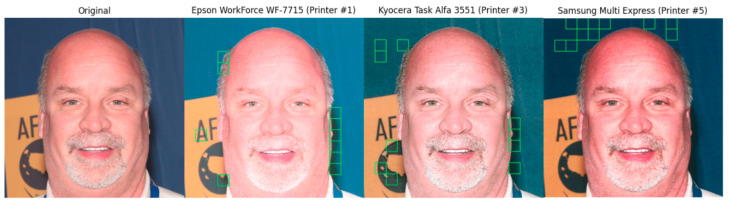
Image 139.jpg of the dataset (first column) and the top 10 energy blocks of size 128 × 128 for different versions of the image printed by various printers (remaining columns).

To extract the ROI patches, we used an approach inspired by the one adopted in [58] to tackle rebroadcast attacks in a data-driven classification scenario. In particular, we extract image patches by firstly applying Canny filter to the whole input image, and then dividing the resulting binary edge image into squared blocks of varying sizes. Then, we calculate the energy *E* of the image patches using the horizontal (*H*), vertical(*V*), and diagonal (*D*) sub-bands of the discrete wavelet transform as follows:

(1) $$E=\frac{\sum_{i=1}^{N}\sum_{j=1}^{N}H{\left(i,j\right)}^{2}+\sum_{i=1}^{N}\sum_{j=1}^{N}V{\left(i,j\right)}^{2}+\sum_{i=1}^{N}\sum_{j=1}^{N}D{\left(i,j\right)}^{2}}{M^{2}},$$

where *N* is the number of values in the sub-bands of DWT and *M* is the fixed size of the squared patches. Afterwards, we ranked the image patches according to their *E* and selected the top 10 energy patches per image. The patches selected in this way compose the RoI subdataset. We chose to calculate the Energy after the binary image is created as we are looking for areas with more edges, instead of those with the highest edge strength. This approach is quite useful when printer noise is hidden in the background or flat areas. For this second set of images, we choose patch sizes of 28×28, 32×32, 64×64, 128×128, 224×224, 227×227, 256×256 and 299×299 in agreement with the most common input formats accepted by the deep learning approaches available today. The RoI subdataset contains, therefore, 128,000 high energy patches. Figure 3 shows some example of high energy patches selected according to the proposed criterion.

### 3.2. VIPPrint Dataset for Synthetic GAN Images Detection

Detecting if an image is a deepfake, i.e., if it has been artificially generated by a GAN, is an increasingly trendy topic in multimedia forensics. In the context of a criminal investigation, for instance, assessing that an image has been taken by a digital camera rather than having been generated artificially can be of fundamental importance to assess the trustfulness of a proof. As another example, in a social media scenario, detecting synthetic images may be useful to understand that a misinformation campaign supported by fake media is ongoing.

So far, research in this area has focused on digital documents, as they are intrinsically linked to fake news in social media. Several strategies have been proposed to deal with such a problem, including analysing the co-occurrence behavior of pixels in RGB channels [39], cross-spectral co-occurrence between pairs of RGB channels [40], discrepancies in color spaces [59], contrastive loss between original and fake images [60] and also other variations of deep learning approaches [38,61]. On the contrary, very few works have considered the detection of deepfake printed images. To date and to the best of our knowledge, the only approach available to deal with the detection of printed manipulated images focuses on the identification of simple manipulations such as Gaussian blurring, Median filtering, resizing and JPEG compression [37]. Yet, printing and scanning back deepfake images is one of the easiest and most effective ways to fool media forensic techniques thought to work in the digital domain.

To promote further research on this topic, we built a second section of the VIPPrint dataset, containing a very large number of natural and GAN-generated face images. Specifically, we printed and scanned a total of 40,000 face images using a Kyocera TaskAlfa3551ci (Printer #3 in Table 1) in the following configurations:

- 16,000 pristine and 16,000 fake images generated by StyleGAN2 [41].
- 3500 pristine and 3500 fake images generated by ProgressiveGAN [42].
- 500 pristine and 500 fake images generated by StarGAN [43].

**Figure 4:**
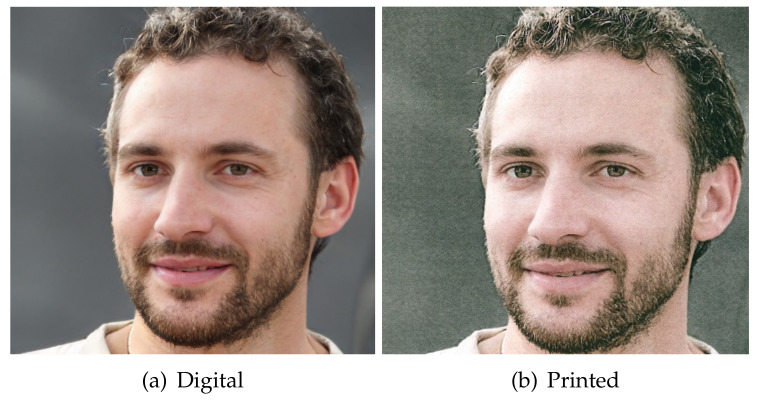
A StyleGAN2 generated image in its original and printed–scanned versions.

**Figure 5:**
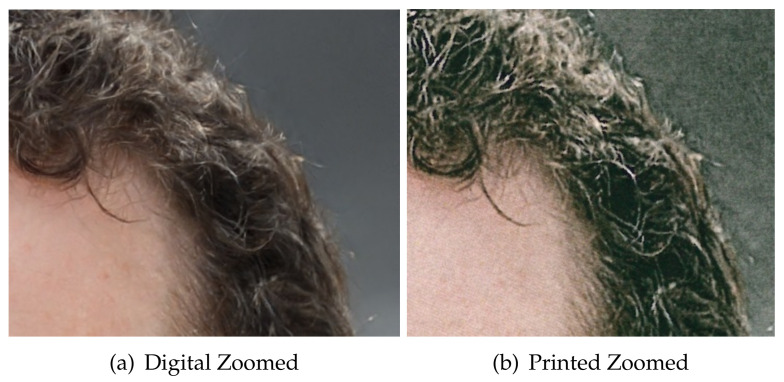
Zoomed regions of the same pictures in Figure 4.

**Figure 6:**
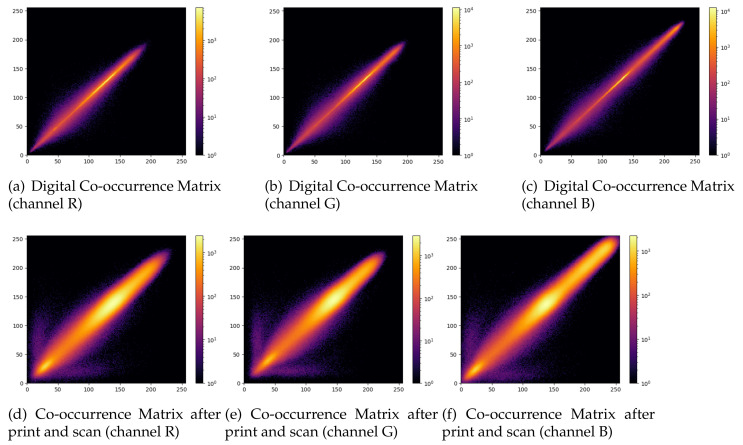
Co-occurrence matrices proposed in [39] to discriminate GAN-generated images from natural ones and their behavior in digital and printed and scanned images: (**top**) GAN image co-occurrence matrices in the digital format (**bottom**); GAN image co-occurrence matrices after printing and scanning.

The first difficulty with these images is the heavy distortion introduced in pixels after printing and scanning. Figure 4 shows how a GAN image is degraded after printing and scanning. The calculated Structural Similarity Index [62] of such images is 0.41 and the Peak Noise to Signal Ratio is 17.65 dB, which corresponds to intense image degradation. The noisy texture of the degradation is visible in the zoomed regions of the digital and printed images highlighted in Figure 5. It is pretty clear from the analysis of this picture that distinguishing between printed pristine and GAN images by looking at textural artifacts only is an extremely difficult task. To further substantiate this hypothesis, in Figure 6 we show the co-occurrence matrices of the RGB bands before and after scanning and printing. The change between the matrices is dramatic, as the image is basically rebroadcasted by another image generation device (i.e., a scanner), possibly erasing the artifacts used to distinguish between natural and GAN images.

**Figure 7:**
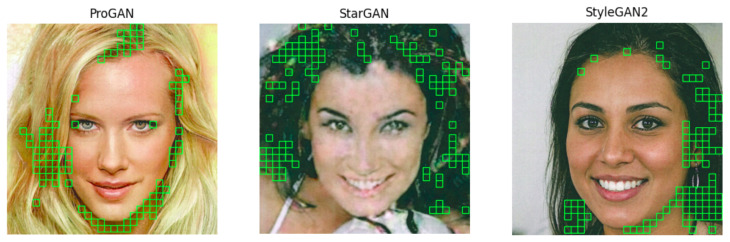
Sample printed pictures from each of the GANs in our dataset with their 64 × 64 top 100 energy blocks.

As for the source attribution dataset, we also built a ROI dataset by applying high energy patch extraction and ranking. However, for this specific problem, the top 100 energy patches were selected (depending on the size of the patches, the selection may correspond to selecting all the patches with non-zero energy). This new subset contained, for the StyleGAN2 case 1109.822 patches for the size 299×299, and 2392.469 patches for the 224×224 size. Patches for other dimensions and GANs can also be extracted by following the same approach. In the rest of the paper, we will focus on StyleGAN2 images, since they are by far the most difficult to discriminate. Figure 7 shows an example of some GAN images of our dataset along with the selected patches.

## 4. Experimental Setup

In this section, we discuss the experimental setup we used to assess the difficulties associated with source attribution (a multiclass classification problem) and GAN image detection (a binary classification problem) on the images of the VIPPrint dataset. Specifically, we present the metrics used for the experiments, the experimental methodology, the statistical tests we adopted (when applicable), and the baseline approaches we tested together with their implementation details.

### 4.1. Metrics

Even if authentication and source linking are different classification problems (i.e., a binary and a multi-class problem respectively), the performance achieved by different methods on such tasks can be measured with similar metrics, by paying attention to interpret them properly according to the considered task. The set of metrics we have used is described in the following.

#### 4.1.1. Recall

For binary classification problems, the recall, also known as true positive rate, indicates the percentage of correctly classified positive samples and is calculated as

(2) $$Recall=\frac{TP}{TP+FN},$$

where TP (True Positives) represents the number of samples correctly classified as positives, and FN (False Negatives) is the number of positive samples wrongly labeled as negative. In our binary classification problem, the Recall metric measures how many GAN images in the testing set were correctly detected as such.

For the multiclass problem of source attribution, a similar metric can be used, with the difference of considering the recall for each class and calculating the weighted mean for all classes as a final metric.

#### 4.1.2. Precision

As a metric complementary to the Recall, we are interested in the classification precision, which is the fraction of correctly classified positives out of all the instances classified as such in a binary classification problem (in our case, GAN images detection). That is

(3) $$Precisiom=\frac{TP}{TP+FN}.$$

For the case of source attribution (a multiclass problem), we considered the precision in a way similar to what we did for the recall. That means, we calculate the precision for each class and then consider the final precision as being the weighted mean of precisions from all classes.

#### 4.1.3. F-Measure

The most important metric for both problems is f-measure (*F*). It measures the harmonic mean of precision and recall and is calculated as follows for the binary classification case:

(4) $$F=2\times \frac{P\times R}{P+R}.$$

For the multi-class source attribution problem, we calculate the f-measure individually for each class by using per-class precisions and recalls and weighting them over all classes, exactly as done for the precision and the recall.

#### 4.1.4. Accuracy

As a final metric, we considered the accuracy. In a binary classification problem, it is defined as the total number of samples correctly classified (in both classes) divided by the number of samples under investigation:

(5) $$Accuracy=\frac{TP+TN}{TP+TN+FP+FN}.$$

In the multiclass problem, we repeated the steps done for other multiclass metrics: we calculated the accuracy per printer and then the weighted average of all the accuracies for all classes was calculated.

### 4.2. Experimental Methodology

To validate the experiments carried out on the VIPPrint dataset, we followed two different approaches, depending on which application we were considering. In the source attribution scenario, we chose the 5×2 cross-validation protocol, as it is considered an optimal benchmarking protocol for machine learning algorithms [63] and was also used in other works on printer attribution [18,34]. According to such a protocol, five iterations of twofold cross-validation were carried out. In other words, the data were firstly randomized, and 50% of data was selected as the training set with the other 50% being used for testing. Then the process was inverted. As stated before, this process was repeated five times (five rounds), resulting in ten experiments of training and testing the machine learning classifiers. Additionally, when using deep learning approaches, we also needed validation data in order to help training. Therefore, we further split the 50% of training data into training data and validation data, with a ratio equal to 70:30.

It is important to notice that, in contrast to camera source attribution validation approaches commonly used in the literature [56,57] that use totally random images generated by different cameras, for source attribution of printed documents, the same document can be printed by different printers [18,34]. In this paper, we consider the source attribution problem as a closed set multiclass problem, where we classify documents printed by known printers in our dataset.

For the GAN-image detection task, we took a set of detectors and trained them on the original digital images, as done in the original papers, and assessed their performance on printed and scanned images. We focus on the detection of the StyleGAN2 images in the VIPPrint dataset, as they are by far the best quality GAN images in the dataset. The procedure we followed to evaluate the performances of the detectors was a simple one: we used 24,000 digital images for training and 6000 digital images for validation, and then we used 2000 printed and scanned images from our dataset for testing the detectors. All the sets were independent and stratified (i.e., images in one set were not present in the others and there was an equal number of images per class).

### 4.3. Statistical Tests

To verify that the source attribution results were statistically significant, we performed a series of two tests in the 5×2 cross-validation procedure. The first one, which we call a pre-test, was used to confirm that all the techniques considered in the experiment are statistically different. If they passed this test, then we did a post test that compared the results in a pairwise manner. The pre-test was done in the distributions of f-measures calculated after ten runs of the 5×2 cross-validation experiments for each technique. The test was applied to an input matrix of *n* rows (where *n* is the number of tested approaches) and ten columns, which were the ten f-measures resulting from the 10 runs. The test aimed at verifying whether the distributions of all the sets of f-measures changed significantly. We used the Friedmann test [64] for this first step, with a confidence level of 95%. In other words, if the calculated p-value was below 0.05, then the null hypothesis, which said that there is no statistically significant difference between the f-measures’ distributions, was rejected and we could move on to the next test.

For the post test, which tested the statistical relevance of each pair of approaches, we considered the Student’s t-test [65]. This test can determine if there is a significant difference between the means of f-measures distributions taken pairwise. To apply this test to our scenario, we also considered the same set of 5×2 f-measure results, but now for each possible pair of approaches. In this test, we set the confidence level to 95%: if the calculated p-value was below 0.05, then the null hypothesis, which stated that there is no statistical significance between the performance of the pair of approaches, was rejected.

### 4.4. Baseline Methods

In this section, we briefly describe the baseline methods considered in our tests.

#### Source Attribution

For this problem, we selected 12 approaches divided into three sets. In the first set, which we call image texture descriptors, we used a set of common descriptors that are mainly used for image characterization. For a source attribution task, such descriptors can be useful to differentiate printers’ banding artifacts efficiently if the analyzed patterns do not change much, and therefore, they normally exhibit good performance in some printer source attribution tasks [18,20,22,34]. We considered four approaches in this set as follows.

- The gray histogram [66] (hereafter referred to as GH) divides the grayscale version of the analyzed image into a fixed number of blocks. Then, a histogram of gray intensities is calculated for each block and all the histograms together are used to generate a description vector.
- The histogram of oriented gradients [67] (hereafter referred to as HOG) extracts the edges in the image by means of the Sobel kernel gradients; then it computes the gradients for all the orientations. Finally, a histogram of such orientations is fed into the input of a machine learning classifier.
- The edge histogram [66] (hereafter referred to as EH) is similar to HOG. However, it calculates, for each block, the dominant edge orientation instead of all of them, and the descriptor is a histogram of these orientations.
- The local binary patterns [68] divide the image into blocks and compare each pixel in a block to all its neighbors. If the pixel in the center of the block is greater than a neighbor’s value, then a 0 digit is written (1 otherwise). Considering eight neighbors in each block, 8-digit binary numbers are generated for each pixel in a block. Such digits are converted to decimals and histograms for each block are calculated, normalized, and concatenated to describe the image.

The second class of approaches has already been introduced in Section 2, and they are referred to as feature based source printer source attribution baseline techniques. These approaches were already validated in the printer source attribution problem by previous works in the literature, and they are:

- The multidirectional version of the gray level co-occurrence matrix (GLCM-MD) from Ferreira et al. [34].
- The multidirectional and multiscale version of the same approach proposed in [34] (GLCM-MD-MS).
- The convolutional texture gradient filter in a 3×3 window [34] (CTGF-3X3).
- The 39 statistical features from the diagonal discrete wavelet transform sub-band from Choi et al. [26] (DWT-STATS).

Finally, the third set of approaches belong to the class of data-driven baselines and rely on the training of deep neural networks. For this set, we considered several convolutional neural network approaches analyzed in [36] for printer source attribution. These are:

- The 16 and 19 layer versions of the VGG convolutional neural network [69] (VGG−16 and VGG−19).
- The 50 and 101 layer versions of the RESNET convolutional neural networks [70] (RESNET-50 and RESNET−101).

#### Printed and Scanned GAN Image Detection

For the deepfake detection task, we chose a set of deep learning classifiers proposed in the literature for digital images. The first three approaches were based on ImageNet dataset pre-trained models and their use for GAN images detection was validated in the work of Marra et al. [38]. They are:

- The Densely connected networks [71] (DENSENET)
- The third version of InceptionNet [72] (INCEPTION-V3);
- The InceptionNet evolution considering fully separable filters [73] (XCEPTION)

The other set of deep neural networks were ad hoc networks designed for the GAN detection problem. These networks act on pre-processed data, namely, the co-occurency matrices of image channels, and they are:

- A CNN that acts on three co-occurence intra-channel matrices [39] (CONET);
- A CNN that acts on six co-occurence matrices considering both intra- and inter-channel co-occurrences [40] (CROSSCONET).

All these five CNNs have been retrained on StyleGAN2 and pristine digital images as described in Section 4.2. In conclusion, we considered 12 baseline methods for the source attribution tasks and 5 for the GAN-image detection task.

### 4.5. Implementation Details

To ensure the reproducibility of our results, we provide all the implementation details we used to achieve our results. We start with the source attribution approaches that we had to re-train from scratch. We had to do that because the eight printers used to build the VIPPrint dataset had never been used before in a source attribution problem. Then, we report the implementation details of the pre-trained baseline models on digital we used to distinguish printed and scanned GAN and pristine images.

We start with the feature engineering approaches. For GH, LBP, EH, HOG, and DWT we used Python implementations, whereas for GLCM-MD, GLCM-MD-MS and CTGF-3X3 we used MATLAB implementations available at the authors’ source code website [74]. Although the implementations used different programming languages, we used them only to extract the features, using a Linear SVM from Python’s sci-kit-learn library (http://scikit-learn.org, accessed on 11 February 2021) for the final classification stage. We chose a linear kernel support vector machines classifier, as it is well suitable to deal efficiently with high-dimensional features. We performed a grid-search approach to find the best parameters to train the classifiers for each of the 10 experiments. This was done by applying a five-fold cross-validation procedure to the training data only. The classifiers’ parameter *C* was varied in the set C={0.1,1,10,100,1000}, and the best value was used to train the classifier.

In contrast to the previous approaches, those based on convolutional neural networks were applied patch-wise, with patches of size 224×224 for VGG−16, VGG−19, RESNET-50, RESNET−101, INCEPTION_NET-V3, and DENSENET and 299×299 for XCEPTION_NET. The final classification result for an image was set to be the mode of the classifications obtained on single patches. This approach is commonly known as majority voting and was also validated in printed document forensics research [18,19,20,22,34]. To choose the patches, we applied the highest-energy procedure already described in Section 3. The only exceptions to this rule were the CONET and CROSS-CONET networks for GAN image detection, which acted on 256×256 co-occurence matrices computed on the entire images. We implemented these techniques by using Python’s Tensorflow (https://www.tensorflow.org/, accessed on 11 February 2021), and Keras (https://keras.io/, accessed on 11 February 2021) libraries.

For a fair comparison of the data-driven approaches, we used the following common procedure to train the neural networks:

1. We fine-tuned the neural networks pre-trained on ImageNet with the input training data (e.g., high energized patches), by initializing the weights with Imagenet pre-trained weights. We tried other procedures, such as fine-tuning, only the tops of the networks (i.e., the fully connected layers) and freezing the other layers, but the results were not worthwhile.
2. In the fine-tuning procedure, we cut off the tops of these networks, replacing them with a layer of 512 fully connected neurons, followed by a 50% dropout layer and a final layer with eight or two neurons, depending on the task.
3. The networks were trained with the steepest gradient descent optimizer [75], with an initial learning rate of 0.01. The learning rate was reduced by a factor of $\sqrt{0.1}$ once the validation loss stagnated after five epochs. We fixed the learning rate lower bound to 0.5 $\times 10^{-6}$. We trained the networks on minibatches of size 32 for source attribution and 16 for GAN detection.
4. We set the maximum number of epochs for source attribution to 300 epochs. However, after 20 epochs we implemented an early stopping procedure if the validation loss did not improve. For deepfake detection, we chose 10 epochs and the early stopping condition was implemented after five epochs, as we were using much more data.
5. We used data augmentation for the source attribution task by using the following image processing operations: rotation, zoom, width shifts, height shifts, shears, and horizontal flips. For GAN detection, since much more training data are available (more than 300,000 images), we did not use any data augmentation.

Finally, all the data presented in this paper, including the two datasets, the scripts for generating the high-energy blocks, 5×2 cross-validation data, and some of the source code used are all available at https://tinyurl.com/vipprint, accessed on 11 February 2021.

## 5. Experimental Results

In this section, we discuss the results of our comparative study for both source attribution and GAN-image detection.

### 5.1. Source Attribution

An overall view of the average results we got for the 12 baseline source attribution techniques we have tested is reported in Table 2.

The first aspect to be noticed in the results shown in Table 2 is the bad performance obtained by methods based on general-purpose texture descriptors. The GH descriptor, for example, tries to discriminate printers by assuming that different printers print the same images using different colors, which is supposed to be seen in different histograms plotted in the *n*-dimensional space and clustered by hyperplanes such as those from the SVM classifiers. That assumption failsm as the resulting f-measure (0.53) is pretty similar to a random guess. The approaches relying more on the effects of gradients and edges (EH and HOG), where the banding and other printing artifacts are more evident [34], achieve slightly better but still poor performance. The best f-measure in this class of techniques was obtained by the LBP descriptor (*F* = 0.75). A possible explanation for the better performance of LBP compared to other texture descriptors is that it explores gradient information by encoding, in several regions, the neighborhood relationships. This can better identify the behavior of printer patterns compared to other texture descriptors.

The second set of techniques included approaches based on handcrafted features specifically tailored for the source attribution problem. To start with, we found that the performance of DWT-STATS (*F* = 0.76) dropped with respect to the performance reported in the original paper [26], highlighting that different datasets with modern printers may confuse such characterization. Additionally, from the discussion done in Section 3, we found that considering statistics from a specific wavelet channel allows identifying different brands, but does not work well when identical devices are included in the set. Other descriptors from [34] show better, but still unsatisfactory results. CTGF-3X3 filters convolutional generated features, building their histogram in a gradient interval. Said approach yielded an average *F* = 0.79, which is considered a good result when compared with the common texture descriptors we considered and discussed in the previous paragraph. We can also see from Table 2 that better performances were also obtained by GLCM-MD (*F* = 0.78) and GLCM-MD-MS (*F* = 0.84). These approaches consider more directions in the neighborhood of pixels and more statistics in the co-occurrence matrices. Additionally, for GLCM-MD-MS, more scales were used in order to achieve invariance with respect to the size of the printed pattern. Such features can be considered ad hoc texture features specific for laser printer attribution, thereby achieving better performance than general texture descriptors.

Finally, the last set of techniques are based on CNNs [36]. Let us consider first the shallower networks, namely, VGG−16 and VGG−19. They provide the two worst results for all metrics, while also showing a very high standard deviation, indicating very unstable training. Two possible reasons for such bad performance are the shallowness of the networks and their very simple architecture, including only convolutional and pooling layers. Those explanations were confirmed by the results gotten by deeper and more complex networks, RESNET-50 and RESNET−101 CNNs. These networks exhibited (by far) the top two results of our tests, with *F* = 0.91 for RESNET-50 and *F* = 0.90 for RESNET−101.

To better investigate the differences between these networks, we start to discuss where they fail and succeed in the printer attribution task. Table 3 and Table 4 show the confusion matrix of these approaches. It can be seen that both approaches have strong difficulties to discriminate two printers from Kyocera: the Color-Laser and a specific Taskalfa model (printers #2 and #3 of our dataset). This result is somewhat surprising because these printers are quite different physically (Taskalfa is a multifunctional printer and Color-Laser is an ordinary laser printer). One possible explanation is that the two printers could have shared some components in their manufacturing process. RESNET-50 showed slightly better performance, as it is less affected by said problem and also because it classified 4 out of 10 printers perfectly, instead of 3 out of 10.

As a final step of the printer attribution experiments, we analyzed the statistical significance of the results. By applying the Friedmann test to 12 vectors (one for each approach) with the 10 f-measures, we got a p-value lower than 0.01, thereby proving that the differences in the f-measures between all the approaches are statistically significant. As a second step, the results of the pairwise statistical tests (Student’s t-test) are shown in Table 5.

The first noticeable behavior in Table 5 is that the large standard deviation of VGG−16 does not allow one to draw statistically significant conclusions for some of the comparisons, namely, those with GH, HOG, EH, and LBP. Other cases where no statistically significant conclusions can be drawn are the comparisons of DWT-STATS and LBP, and HOG with EH.

Finally, we notice that the superior performances of RESNET-50  and RESNET−101 were confirmed by the results of the Student’s t-tests with all the other methods. At the same time, the difference between the performances of these two networks was not statistically significant. Based on these observations, we can conclude that RESNET-50 and RESNET−101 represent the better solutions for the source attribution problem, even though their best performance, with an f-measure equal to 0.91, along with the difficulties in distinguishing some Kyocera printers, leave room for further improvement.

### 5.2. Detection of GAN Images

**Figure 8:**
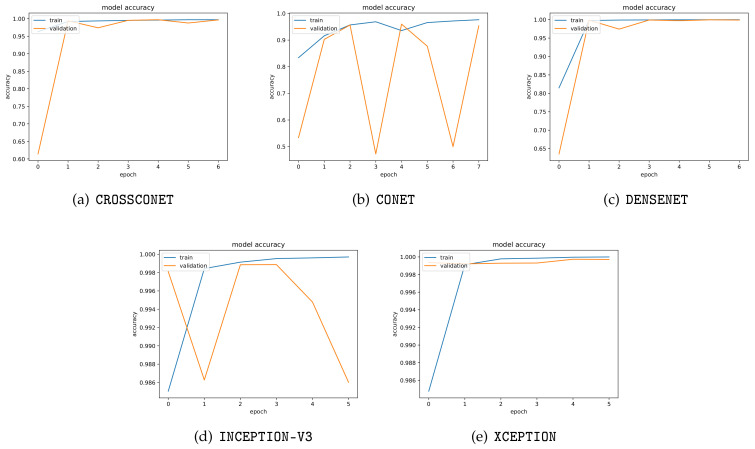
Models of training and validation curves when applied to digital images.

We now discuss the results of GAN image detection on printed and scanned documents. For that, we considered a set of CNNs trained on different patch sizes (i.e., 224×224 and 299×299) with majority voting and also 256×256 co-occurrence matrices without majority voting. We first show, in Figure 8, the training and validation behavior of these networks considered for this experiment when trained on digital images.

Figure 8 shows the training and validation curves’ behavior for INCEPTION-V3 and CONET CNNs. The different patterns visible in the figure can be explained by the different complexity of the architectures we used, the number of layers, and the diversity of training data: INCEPTION-V3 has a very simple architecture with some inception modules in 48 layers, whereas CONET was trained on RGB co-occurrence matrices with seven layers only and with 10 times less data. Those issues notwithstanding, it is expected that, without the early stopping criterion on such a low number of epochs as we used for our CNNs’ training, the curves’ behavior would stabilize after some iterations. However, even with these irregular training curves, the early stopping criterion required less than 10 epochs to be fulfilled, and all models’ training and validation accuracies selected for further testing were higher than 95% when classifying digital images.

As anticipated in Section 3.2, the print and scan process eliminated most of artifacts commonly found on digital images, being the simplest but most efficient attack against such approaches. In Table 6, we show the classification results considering both digital and printed and scanned test images. For the digital case, all the approaches achieved an accuracy higher than 95%, with the worst approach being CONET with 96% accuracy. The CROSSCONET [40] showed better performance than CONET for digital images, as it also looks for artifacts in cross-band co-occurrence matrices. The best approaches in the digital scenario were DENSENET, INCEPTION-V3, and XCEPTION, with virtually perfect results. The power of ROIs majority voting is exemplified by the confusion matrix of the XCEPTION CNN in Table 7. It can be seen from that table that the approach misclassifies only seven 299×299×3 high-energy testing patches, explaining the perfect classification after majority voting.

When faced with printed and scanned images, though, all methods failed, as can be seen on the rightmost part of Table 6. These results confirm that most of the artifacts used by the detectors to distinguish between GAN and real images, such as warping, blur, noise, correlation, and image statistics, are gone when images are printed and recaptured. In fact, all the approaches provided accuracy equal to 0.5 with zero precision and recall, meaning that all the images were classified as natural ones. The only minor exception is represented by CROSSCONET, which correctly classified 21% of StyleGAN2 images as fake images, as can be seen from the confusion matrix shown in Table 8.

The poor results obtained when GAN-image detectors were trained on digital images and applied to printed and scanned images call for new research on this topic, in order to face the fact that counterfeiters could print and scan fake images in order to avoid being revealed as such. We are, therefore, confident that the availability of the VIPPrint dataset will help researchers to solve this challenging task.

## 6. Conclusions

The accessibility and constant upgrading of devices capable of generating high-quality physical documents have raised the necessity for forensic methods to attest to the reliability of printed documents and possibly link illegal or criminal documents to their creators. Authentication and source linking of printed documents may also have huge economic impacts, since it may help to tackle the diffusion of counterfeited products. Although several works in the scientific literature have addressed said issue, all of them failed in two aspects: (i) they did not consider a dataset that grows with time, including more recent and professional printing devices; and (ii) they did not consider the authentication of printed artificial images.

In this paper, we showed the extent of such limitations by validating existing authentication and source linking methodologies on a novel dataset specifically made for printed document forensics. The new dataset, the VIPPrint dataset, presents the first version of an ongoing effort to build a challenging environment for printed image forensics. To the best of our knowledge, the dataset contains the richest publicly available corpus of printed natural and artificial images, with 40,000 images addressing deepfake face-image detection, and 1600 images focusing on source attribution in a closed set of eight printer sources. The experiments we have run showed that this dataset results in an error probability of at least 9% for the best baseline source attribution methods. The dataset raises even more challenging problems in the case of GAN-image detection, given that StyleGAN2 images look like the original ones for all the tested methods after they are printed and scanned.

The experiments we ran have guided us toward a lot of further work. First of all, we will continue updating the dataset to include new printers, more scanners, other GANs, and acquisition devices such as digital single lens reflex (DSLR) cameras. Second, we aim at investigating novel ways of selecting regions of interest in the digitized images and also considering other color spaces in addition to RGB. Finally, we are also headed toward investigating and applying adversarial attacks in the printed domain; we will add relevant features to our dataset in order to evaluate the effectiveness of printed document forensic methods.

## Figures and Tables

**Table 1:** A list of eight laser printers that compose the first version of the VIPPrint dataset.

VIPPrint Dataset- Printer Source Linking					
ID	Brand	Model	Resolution	Type	#Images
**#1**	Epson	WorkForce WF-7715	4800 × 2400 dpi	Laser	200
**#2**	Kyocera	Color Laser	600 × 600 dpi	Laser	200
**#3**	Kyocera	TaskAlfa 3551	600 × 600 dpi	Laser	200
**#4**	Kyocera	TaskAlfa 3551	600 × 600 dpi	Laser	200
**#5**	Samsung	Multiexpress X3280NR	600 × 600 dpi	Laser	200
**#6**	HP	Color LaserJet Pro rfp-r479fdw	600 × 600 dpi	Laser	200
**#7**	HP	Color LaserJet rfp-r377dw	600 × 600 dpi	Laser	200
**#8**	OKI	C612 LaserColor	1200 × 600 dpi	Laser	200

**Table 2:** Average performance for the source attribution problem. The approaches are divided by category, and boldfaced entries denote the solutions specifically designed for the source printer attribution problem. The best results for each metric are highlighted in yellow.

Type	Method	5 × 2 Cross Validation Results–Close Set Printer Attribution			
		Input Size	F	Precision	Recall
TEXTURE DESCRIPTORS	GH [66]	Image	0.52 ± 0.01	0.53 ± 0.01	0.52 ± 0.01
	HOG [67]	Image	0.68 ± 0.01	0.69 ± 0.01	0.68 ± 0.01
	EH [66]	Image	0.69 ± 0.01	0.69 ± 0.01	0.69 ± 0.01
	LBP [68]	Image	0.75 ± 0.01	0.75 ± 0.01	0.75 ± 0.01
**FEATURE-BASED BASELINES**	**DWT-STATS** [26]	**Image**	**0.76 ± 0.01**	**0.76 ± 0.01**	**0.76 ± 0.01**
	**GLCM-MD** [34]	**Image**	**0.78 ± 0.01**	**0.79 ± 0.01**	**0.78 ± 0.01**
	**GLCM-MD-MS** [34]	**Image**	**0.84 ± 0.01**	**0.84 ± 0.01**	**0.84 ± 0.01**
	**CTGF-3x3** [34]	**Image**	**0.79 ± 0.01**	**0.79 ± 0.01**	**0.79 ± 0.01**
**DATA-DRIVEN BASELINES**	**RESNET-50** [36,70]	**224 × 224 patches**	**0.91 ± 0.01**	**0.92 ± 0.00**	**0.91 ± 0.00**
	**RESNET-101** [36,70]	**224 × 224 patches**	**0.90 ± 0.01**	**0.92 ± 0.00**	**0.91 ± 0.00**
	**VGG−16** [36,69]	**224 × 224 patches**	**0.46 ± 0.45**	**0.45 ± 0.46**	**0.51 ± 0.40**
	**VGG−19** [36,69]	**224×224 patches**	**0.37 ± 0.44**	**0.37 ± 0.44**	**0.42 ± 0.39**

**Table 3:** Confusion matrix of RESNET-50 for the source attribution problem.

Confusion Matrix – RESNET50								
**Printer**	**Epson-WorkForce** **WF-7715**	**Kyocera** **ColorLaser**	**Kyocera** **TaskAlfa3551ci**	**Kyocera** **TaskAlfa3551ci-2**	**Samsung** **Multiexpress** **X3280NR**	**HP** **Color-LaserJet** **Pro** **rfp-r479fdw**	**HP** **Color-LaserJet** **rfp-r377dw**	**OKI-C612** **LaserColor**
**Epson-WorkForce** **WF-7715**	100.00%							
**Kyocera** **ColorLaser**		56.00%	41.00%	3.00%				
**Kyocera** **TaskAlfa3551ci**		5.00%	92.00%	3.00%				
**KyoceraTaskAlfa** **3551ci-2**		1.00%		99.00%				
**Samsung** **Multiexpress-X3280NR**					100.00%			
**HP** **Color-LaserJet** **Pro** **rfp-r479fdw**						98.00%	2.00%	
**HP-Color-LaserJet** **rfp-r377dw**							100.00%	
**OKI-C612** **LaserColor**								100.00%

**Table 4:** Confusion matrix of RESNET−101 for the source attribution problem.

Confusion Matrix – RESNET101								
**Printer**	**Epson-WorkForce** **WF-7715**	**Kyocera** **ColorLaser**	**Kyocera** **TaskAlfa3551ci**	**Kyocera** **TaskAlfa3551ci-2**	**Samsung** **Multiexpress** **X3280NR**	**HP** **Color-LaserJet** **Pro** **rfp-r479fdw**	**HP** **Color-LaserJet** **rfp-r377dw**	**OKI-C612** **LaserColor**
**Epson-WorkForce** **WF-7715**	100,00%							
**Kyocera** **ColorLaser**		35.00%	60.00%	5.00%				
**Kyocera** **TaskAlfa3551ci**		5.00%	93.00%	2.00%				
**KyoceraTaskAlfa** **3551ci-2**			1.00%	99.00%				
**Samsung** **Multiexpress-X3280NR**					100.00%			
**HP** **Color-LaserJet** **Pro** **rfp-r479fdw**						98.00%	2.00%	
**HP-Color-LaserJet** **rfp-r377dw**						1.00%	99.00%	
**OKI-C612** **LaserColor**								100.00%

**Table 5:** Pairwise statistical comparison between different source attribution techniques.

Method	GH [66]	HOG [67]	EH [66]	LBP [68]	DWT-STATS [26]	GLCM-MD [34]	GLCM-MD -MS [34]	CTGF-3x3 [34]	RESNET-50 [36,70]	RESNET−101 [36,70]	VGG−16 [36,69]	VGG−19 [36,69]	TOTAL
**GH** [66]	0	−1	−1	−1	−1	−1	−1	−1	−1	−1	0	0	−9
**HOG** [67]	1	0	0	−1	−1	−1	−1	−1	−1	−1	0	1	−5
**EH** [66]	1	0	0	−1	−1	−1	−1	−1	−1	−1	0	1	−5
**LBP** [68]	1	1	1	0	0	−1	−1	−1	−1	−1	0	1	−1
**DWT-STATS** [26]	1	1	1	0	0	−1	−1	−1	−1	−1	1	1	0
**GLCM-MD** [34]	1	1	1	1	1	0	−1	0	−1	−1	1	1	4
**GLCM-MD-MS** [34]	1	1	1	1	1	1	0	1	−1	−1	1	1	7
**CTGF-3x3** [34]	1	1	1	1	1	0	−1	0	−1	−1	1	1	4
**RESNET-50** [36,70]	1	1	1	1	1	1	1	1	0	0	1	1	10
**RESNET−101** [36,70]	1	1	1	1	1	1	1	1	0	0	1	1	10
**VGG−16** [36,69]	0	0	0	0	−1	−1	−1	−1	−1	−1	0	0	−6
**VGG−19** [36,69]	0	−1	−1	−1	−1	−1	−1	−1	−1	−1	0	0	−9
*1 = Line method is better than column method*													
*0 = Line method is equivalent to column method*													
*−1 = Line method is worse than column method*													

**Table 6:** Results of GAN image detection tests for digital (left) and printed and scanned images (right). Best results are highlighted in yellow

			Digital Images Testing Results				Printed and Scanned Testing Images Results			
Method	Training/Validation Data	Input Size	Acc	F	Precision	Recall	Acc	F	Precision	Recall
**DENSENET** [38,71]	Images Patches	224 × 224 × 3	1.00	1.00	1.00	1.00	0.50	0.00	0.00	0.00
**INCEPTION-V3** [38,72]	Images Patches	224 × 224 × 3	1.00	1.00	1.00	1.00	0.50	0.00	0.00	0.00
**XCEPTION** [38,73]	Images Patches	299 × 299 × 3	1.00	1.00	1.00	1.00	0.50	0.00	0.00	0.00
**CONET** [39]	Co-occurency Matrices	256 × 256 × 3	0.96	0.96	0.95	0.98	0.50	0.00	0.00	0.00
**CROSSCONET** [40]	Co-occurency Matrices	256 × 256 × 6	0.99	0.99	0.98	1.00	0.50	0.29	0.50	0.21

**Table 7:** Confusion matrix of XCEPTION 299×299×3 patches classified for digital GAN-image detection.

Confusion Matrix Xception Digital Test Patches		
**Class**	**Real**	**Fake**
**Real**	**8914**	5
**Fake**	2	**8983**

**Table 8:** Confusion matrix of CROSSCONET for printed and scanned GAN-image detection.

Confusion Matrix CROSSCONET PrintScan Test Images		
**Class**	**Real**	**Fake**
**Real**	**796**	204
**Fake**	788	**212**

## Data Availability

Our data is available on a public repository. The address is informed in the paper (http://tinyurl.com/vipprint, accessed on 11 February 2021). The DOI for our dataset is http://doi.org/10.5281/zenodo.4454971 , accessed on 11 February 2021.
